# On the Secreting Function of the Colon

**Published:** 1851-02

**Authors:** 


*On the Secreting Function of the Colon.* By Jas. Paul, M. D. Read before the District Medical Society for the County of Mercer.

Although great and deserved attention is paid to the secretions in disease, both urinary and fæcal, and in a great many of the diseases to which the human frame is liable, particularly in fevers, there is no surer criterion to lead us in our prognostications, or guide us in our remedial efforts than the appearance of the excretions. I do not know if we are so thoroughly acquainted with the philosophy of the fæcal discharges as we ought to be, or that we view them altogether in the physiological bearing to which they properly belong in the animal economy.

We are, I think, too much in the habit of viewing the excrements merely as an index of the food having undergone the proper and necessary process of digestion, and when we see pieces of undigested aliment mixed up in the fæces, we naturally conclude and say that the substances consumed, whether of potato, apple, carrot, or whatever else has been partaken of, has not been digested.

Even this, however, is not without its use—for although in such cases the pressing symptoms, whether of croupy cough, nervous twitches, or convulsive spasms, are relieved by evacuating the alimentary canal of foreign and irritating substances, it yet enables us to note what portion of the digestive function is incomplete, whether the deficiency lies in the non-rendering the vegetable food into the saccharine principle, or otherwise, and so to alter the food to that which can be digested, and direct our remedial efforts to that portion of the function which is deficient.

My purpose at this time, however, is not with the function of digestion, but to direct our attention to the fæcal secretions or excretions, and to the colon, or large intestine, as a great secreting organ,

Every practitioner is more or less acquainted with the appearance of the secretions as they are passed from the body of a patient laboring under fever—the brownish watery discharges having a cadaverous or fleshy smell, the black or dark green discharges resembling blubber, or the green fat of turtle, having a highly offensive and putrid odor—and the gradual return to the yellowish watery discharges having as convalescence is established, more consistence, and the more genial odor of proper fæces. I do not intend to enter into, neither is it needful that I should, the various appearances of the fæcal discharges in disease, nor the altered appearances caused by various remedial agents.

It has been a question among physiologists of the older school, whether absorption takes place in the larger intestines? On this subject, Blumenbach has the following—"It has been inquired whether lacteals exist also in the large intestines, and their existence has been contended for from the effects of particular injections, nutrients, inebriating, &c., and also by the circumstance that the fæces if retained for any length of time become hard and dry. Although these arguments do not demonstrate the absorption of genuine chyle below the valve of Fallopius, nevertheless, it is rendered probable by the visible existence of an abundance of lymphatics in the large intestines having the same structure and function with the lacteals, for these absorb lymph from the intestines during the absence of chyle.

"But the very different structure of the internal coat of the large intestines from that of the villous coat of the small, strongly argues that they are not naturally intended to absorb chyle."—*Blumenbach*, 233.

Our present views of the transudation of liquids through animal texture, will readily enable us to comprehend how absorption may take place, and nourishment be conveyed into the system when thrown into the large intestines, and even only into the rectum by means of injections. Nor is it at all incompatible with physiological facts that absorption and secretion should go on in the same organ, and through the same texture by different sets of vessels.

The same unsatisfactory knowledge, if I may be allowed the expression, exists regarding the functions and uses of the *mesenteric glands* of the colon. Prof. Grant, treating of these organs, says: "There are nearly a hundred of these organs on the human lacteals, and about a fourth part of these belong to the colon; but the changes they effect on the fluids which are incessantly passing through them during life, and even for some time after death, or the uses to which they are subservient in the economy, are still unknown, like the functions of many other obvious parts of our most complicated and wonderful fabric."—*Prof. Grant's Lectures, Jan.* 26, 1824.

Following up the argument of the absorption of chyle, and its having been seen in the mesenteric veins, Blumenbach says: "The assertion that chyle has been seen in the mesenteric veins requires further investigation and proof; so that I cannot believe that they carry anything more than blood, being *carbonized* and destined for the formation of bile.—*Blumenbach*, 234.

* That these derangements occur in fever, is often owing to injudicious cathartization.—Editor Buffalo Medical Journal.

Here, then, we find the blood loaded and surcharged with that principle, of which a great portion of the fæces is composed.

Having thus briefly alluded to the views generally and formerly entertained by physiologists, let us enter more minutely into the structure of that portion of the large intestines in which this most important function is situate. "A part of the fæces, however—says Carpenter,—may be derived from the secretions of the enteritic mucous membrane, and of its glandulæ; the surface of the former, with its simple follicles, probably secretes nothing but mucus; but the glandulæ with which it is so thickly studded appear to serve as the channel for the elimination of putrescent matter from the blood. There can be no doubt that a large quantity of fluid is poured out by these glandulæ when they are in a state of irritation from disease, or from the stimulus of a purgative medicine; since the amount of water discharged from the bowels is often much greater than that which has been ingested, and must be derived from the blood."—*Carpenter*, 501.

For a description of these glandulæ, allow me to transcribe from the same author the following: "The whole mucous surface of the intestinal canal is furnished with glandular follicles of a very similar character; of which some approach those of the stomach in complexity of structure, whilst others evidently correspond with the crypts of ordinary mucous membrane. An innumerable multitude of pores are easily seen by the aid of a simple lens to cover the whole internal surface of the large intestines, and these are the entrances to tubular follicles closely resembling those of the stomach, but more simple in structure. Their cœcal extremities shut against the submucous tissue; towards the end of the rectum, however, they are much prolonged, and constitute a peculiar layer between the mucous and muscular coats; the tubes which are there visible to the naked eye being erect, parallel, and densely crowded. These glands probably form the peculiarly thick and tenacious mucus of the large intestine."—*Carpenter*, 668.

And of the functions of this glandular structure, the same author observes, "Although the particular use of each variety of the intestinal glandulæ cannot yet be determined, there seems little doubt that their general function is to eliminate from the blood those putrescent matters which would otherwise accumulate in it; whether as one of the results of the normal waste of the system, or as produced by various morbific causes which act as *ferments*, and thus occasion an unusual tendency to decomposition in the solids and fluids of the body. That the putrescent elements of the fæces are not derived from the food taken in, so much as from the excreting action of the intestinal glandulæ, appears from this consideration among others; that fæcal matter is still discharged, even in considerable quantities, long after the intestinal tube has been completely emptied of its alimentary contents. We see this in the course of many diseases where food is not taken for many days, during which time the bowels have been completely emptied of their previous contents by repeated evacuations, and whatever then passes in addition to the biliary and pancreatic fiuids must be derived from the intestinal walls themselves. Sometimes a copious flux of putrescent matter continues to take place spontaneously, whilst it is often produced by the agency of purgative medicine. The 'Colliquative Diarrhœa' which frequently comes on at the close of exhausting diseases, and which usually precedes death by starvation, appears not to depend so much upon a disordered state of the intestinal glandulæ themselves, as upon the general disintegration of the solids of the body, which calls them into extraordinary activity for the purpose of separating the decomposing matter."—*Carpenter*, 670.

What I have just read is so comprehensive, and brings the subject so forcibly and powerfully to the mind as to preclude the necessity almost of entering more fully upon it.

My attention was particularly drawn to this subject by the frequent occurrence of immense quantities of the morbific and putrid discharges by stool, in tropical fevers, immediately before returning convalescence. At the commencement of the disease, the alimentary canal would be carefully emptied by repeated doses of purgative medicine, the fever would continue, watery stools would supervene; at this period the patient would take the simplest nourishment, and that in small quantities, and in many cases none at all, the stomach rejecting every particle of food exhibited—in the progress of the disease, the patient prostrated and nearly fainting on the least exertion, large dejections would occur of dark-colored gelatinous offensive matter—quarts, and I may say gallons on some occasions, are passed off at repeated operations—and although the patient at this time would be scarcely able to move or speak, yet after such evacuations he would feel more easy—a moisture appear on the surface—the critical moment being seized, and nourishment with wine or brandy exhibited—the patient slumbers, and from that time convalescence progresses.

And what is the result if this dark offensive matter is not thrown off? It is more than probable that the fever will continue, and in more favored climates a slow and dilatory convalescence may ensue, or the whole system becomes corrupted, and *in a tropical climate* putrefaction succeeds almost ere the being has ceased to breathe.

In the epidemic which has so lately made such havoc and ruin in its course in some cities of the Union, causing such fearful mortality, the non-performance of the proper functions of the secreting glands of the intestines is no doubt a principal effect. Without entering into the manner in which the morbific poison of the cholera acts on the system, we see an abeyance of the proper secretions—of bile, urine, and fæcal discharges, and in their stead a watery secretion is ejected, even with force, from the stomach and intestines, without straining, and without pain; indeed, so offensive is the presence of this secreted fluid to the stomach and intestinal canal, that the patient can scarcely control its ejectment for a few seconds. And this unusual parting with the serous portion of the blood leaves the remaining portion thick, viscial, and incapable of entering the minute or capillary vessels, and collapse is the consequence—but arrest the serous discharges, and once produce a fæcal evacuation with tinges of biliary secretion, and there is every chance of the recovery of the patient. Hence, it is obvious that the secreting organ of the large intestines is seriously affected in this formidable disease. I call it formidable from the fatality attending the visitation, but in my opinion controllable in a great majority of cases where the patient has been timely put under the care of the physician, and remedial and energetic measures have been pursued.

Every practitioner will no doubt bring to his recollection cases in which the patient, even after repeated and free evacuations, will answer to the inquiry regarding his feelings, "I am better—my medicine has acted very well—still I feel as if there was yet something to come away." Is it not probable that this feeling, indescribable to the patient, not amounting to pain, and relieved by a copious evacuation, has been owing to the surcharged state of the mesenteric veins, and the relief the consequence of the active secretion from the *glandulæ* which has been the subject of consideration.

The secretion of the liver is looked for, and the returning appearance of bile in the fæcal discharges is held in great estimation, and properly so, by most, if not by all practical physicians; its proper action is absolutely necessary to recovery from disease and the enjoyment of health. It is not my object to withdraw attention from that most important organ, but to direct more particular attention to the colon as a great secreting organ; that the fæces, which in health may contain that portion of the food which has not been absorbed into the system, more especially when a superabundant quantity of aliment has been consumed, is for the most part secreted by the large intestines, from which the deleterious and disintegrated portions of the organic mass is passed away, and the system freed of much of the superabundant carbon which may not be required for the purposes of respiration. *New Jersey Medical Reporter.*

The study of the functions of the large intestine is to be commended to those practitioners, who, in the language of Dr. Stokes, (which is more significant than chaste,) derive facts for diagnosis, and the indications for treatment, chiefly from the inspection of chamber pots. Some Physicians appear to see in the varied appearances of the dejections, little else than different conditions of the secretions of the Liver. Indeed, the *Hepatic pathology* of modern days, now happily on the wane, has been mainly instrumental in giving to the alvine evacuations undue relative importance as criteria for discriminating and treating diseases. The secretions from the liver in fact constitute only an element, and, probably, a minor element in producing both the healthy and morbid appearances of the dejections from the bowels. It may be doubted if the *bilious stools* which often appear to afford so much satisfaction, have more than a remote dependence on the presence of bile. The changes in the color, consistence, odor, etc., of the feces depend mainly on the function of fecation, performed by the large intestine, with which function the bile is concerned only as one of the collateral circumstances involved. As furnishing symptoms by which to estimate the condition of the organism, and the phases of disease, of how much greater importance is the secretion of urine, than the intestinal excrement! And, yet, how seldom is the former studied and observed with care, while the latter not infrequently seems to absorb both the senses and the meditations of the practitioner! In the investigation of many diseases the alvine discharges possess but little real relevancy. In cases of diseases of the chest, or heart, for examples, how absurd for the dejections to be carefully reserved and assiduously inspected, while physical exploration is depreciated or neglected! But it has fallen to our lot, as it must also to that of our readers, to witness illustrations of this absurdity. The popular mind has unfortunately become imbued with the exaggerated importance of seeing and smelling every thing that passes the bowels in all cases of sickness, without reference to the seat and character of the malady. The comfort of the medical practitioner will be in no small degree promoted by the diffusion of correct notions respecting the function of fecation, and a consequent abatement of that popular error which regards attention to this portion of the animal economy as a *sine qua non* under all circumstances. Far be it from us to advocate any neglect of the *pot de chambre,* but as an optical instrument for developing useful information, we fancy it resembles the *kaleidoscope,* oftener than the *camera obscura.*

EDITOR BUFFALO MEDICAL JOURNAL.

